# Measurement of urinary collagen cross-links indicate response to therapy in patients with breast cancer and bone metastases

**DOI:** 10.1038/sj/bjc.6690496

**Published:** 1999-06

**Authors:** J Walls, A Assiri, A Howell, E Rogers, W A Ratcliffe, R Eastell, N J Bundred

**Affiliations:** Department of Surgery, University Hospital of South Manchester, Nell Lane, Manchester, M20 8LR, UK; Department of Human Metabolism and Clinical Biochemistry, Northern General Hospital, Herries Road, Sheffield, S5 7AU, UK; CRC Department of Medical Oncology, Christie Hospital, Wilmslow Road, Manchester, M20 4BX, UK; Wolfson Research Laboratories, Department of Clinical Chemistry, Queen Elizabeth Medical Centre, Birmingham, B15 2TH, UK

**Keywords:** breast cancer, pyridinoline cross-links, bone metastases

## Abstract

Objective assessment of response in bone metastases from breast cancer using radiological techniques takes up to 6 months of treatment to be certain of a response, and sclerotic metastases are not evaluable. Standard serum and urinary tumour markers may not always be utilized to predict response, as they may not be elevated, and therefore may not change on treatment. The development of the urinary pyridinoline cross-link assays which measure mature bone breakdown products have been shown to be highly sensitive and specific as a measure of bone change in osteoporosis. We have measured pyridinoline (Pyr) and deoxypyridinoline (Dpyr) cross-links sequentially in 36 breast cancer patients with bone metastases, to determine if the measurement of these analytes predicts response at an earlier stage than radiological assessment. Response was assessed by UICC criteria. Seventeen women responded to hormonal therapy, whilst 19 developed progressive disease. Both Pyr and Dpyr increased sequentially in women with progressive disease with changes becoming apparent by 8 weeks (*P* < 0.03). In responding women, cross-link levels did not change significantly. Pyr and Dpyr were more sensitive and specific than the standard serum tumour marker CA 15-3. Urinary cross-link measurements provide a novel objective method of assessing response to treatment in women with bone metastases. Initial elevated urinary cross-link markers identify patients who tend not to respond to changes in hormonal therapy. © 1999 Cancer Research Campaign


British Journal of Cancer (1999) 80(8), 1265–1270
© 1999 Cancer Research Campaign
Article no. bjoc.1999.0496

### Measurement of urinary collagen cross-links indicate response to therapy in patients with breast cancer and bone metastases

**J Walls[1], A Assiri[2], A Howell[3], E Rogers[1], WA Ratcliffe[4], R Eastell[2] and NJ Bundred[1]**

[1]Department of Surgery, University Hospital of South Manchester, Nell Lane, Manchester M20 8LR, UK; [2]Department of Human Metabolism and Clinical Biochemistry, Northern General Hospital, Herries Road, Sheffield S5 7AU, UK; [3]CRC Department of Medical Oncology, Christie Hospital, Wilmslow Road, Manchester M20 4BX, UK; [4]Wolfson Research Laboratories, Department of Clinical Chemistry, Queen Elizabeth Medical Centre, Birmingham B15 2TH, UK

**Summary** Objective assessment of response in bone metastases from breast cancer using radiological techniques takes up to 6 months of treatment to be certain of a response, and sclerotic metastases are not evaluable. Standard serum and urinary tumour markers may not always be utilized to predict response, as they may not be elevated, and therefore may not change on treatment. The development of the urinary pyridinoline cross-link assays which measure mature bone breakdown products have been shown to be highly sensitive and specific as a measure of bone change in osteoporosis. We have measured pyridinoline (Pyr) and deoxypyridinoline (Dpyr) cross-links sequentially in 36 breast cancer patients with bone metastases, to determine if the measurement of these analytes predicts response at an earlier stage than radiological assessment. Response was assessed by UICC criteria. Seventeen women responded to hormonal therapy, whilst 19 developed progressive disease. Both Pyr and Dpyr increased sequentially in women with progressive disease with changes becoming apparent by 8 weeks ($P < 0.03$). In responding women, cross-link levels did not change significantly. Pyr and Dpyr were more sensitive and specific than the standard serum tumour marker CA 15-3. Urinary cross-link measurements provide a novel objective method of assessing response to treatment in women with bone metastases. Initial elevated urinary cross-link markers identify patients who tend not to respond to changes in hormonal therapy.

**Keywords**: breast cancer; pyridinoline cross-links; bone metastases

Bone metastases are a common clinical problem, affecting 70% of women with breast cancer (Coleman and Rubens, 1987). Difficulties with objective assessment of response to treatment by conventional radiological means, particularly in women with sclerotic or mixed lytic-sclerotic metastases, have led to the investigation of biochemical markers of bone metabolism as potentially useful indicators of disease response (Coleman et al, 1992). Urinary hydroxyproline excretion is the most studied marker of bone resorption but is not specific for bone collagen breakdown and is affected by changes in diet as well as soft tissue destruction by disease (Robins et al, 1982; Moro et al, 1993). Urinary calcium excretion is normal in patients with predominantly sclerotic disease and in patients whose tumours produce parathyroid hormone-related protein (PTHrP), which increases renal tubular reabsorption of calcium (Coleman et al, 1992; Sano et al, 1994).

Pyridinoline (Pyr) and deoxypyridinoline (Dpyr) are cross-links between collagen molecules, and are broken down and released during bone resorption (Fujimoto, 1980; Eyre, 1992). Excretion of the cross-links is unaffected by diet. Dpyr is only found in bone and dentine collagen and thus its excretion is potentially specific for bone collagen breakdown (Robins, 1982). Preliminary results suggest that Dpyr excretion has significant advantages over hydroxyproline as a urinary marker of bone resorption in osteoporotic patients (Eastell et al, 1990; Delmaset al, 1991; Paterson et al, 1991).

Urinary excretion of cross-links has been reported to be elevated in patients with breast and prostate cancer (Sano et al, 1994) and to fall when bone metastases are treated by anti-resorptive drugs such as bisphosphonates (Coleman et al, 1992).

The aim of this study was to assess the ability of two urinary cross-link markers to predict response to hormonal treatment in patients with breast cancer bone metastases. These analytes were measured serially and compared with serum levels of the breast tumour-associated antigen CA15-3 (Robertson et al, 1991) and standard International Union Against Cancer (UICC) radiological criteria of response (Hayward et al, 1977).

### PATIENTS AND METHODS

### Patients

Thirty-six women with breast cancer presenting with metastatic disease in the skeleton were prospectively studied. At relapse or progression of their disease, women were either being treated with adjuvant tamoxifen 20 mg day$^{-1}$ ($n$ = 13), megesterol acetate ($n$ = 3), radiotherapy induced ovarian ablation ($n$ = 1), or on no treatment ($n$ = 19). Four patients presented synchronously with bone metastases and a palpable breast lump.

The diagnosis of bone metastases in all women was made by a radioisotope bone scan at presentation combined with plain

*Received 19 December 1997*
*Revised 5 November 1998*
*Accepted 21 January 1999*

*Correspondence to*: NJ Bundred radiographs of the chest, pelvis and lumbar spine. Radiographs were also taken of any areas of activity on the radioisotope scan or any clinically symptomatic areas of the skeleton. Twelve patients had disease in other sites (soft tissue, $n$ = 8; lung, $n$ = 7; liver, $n$ = 2; brain, $n$ = 1) (Table 1). Sequential radiological monitoring was performed with plain radiographs of the chest, pelvis, lumbar spine and any other affected bones every 3 months. Response to treatment for patients with lytic metastases was assessed by standard radiological UICC criteria at 3 and 6 months, or at disease progression (Hayward et al, 1977). The radiological response to treatment was assessed independently by a consultant radiologist and confirmed by a medical oncologist without knowledge of the cross-link results. Patients with an increase in the number of metastases, or size of metastases, or the development of further lytic metastases were regarded as having progressive disease.

Patients with solely sclerotic metastases were not evaluable according to UICC criteria and were excluded from the study. However, patients with mixed sclerotic and lytic disease, or patients with sclerotic metastases who had clearly evaluable areas of soft tissue disease were included.

For analysis, patients with responding or stable disease for more than 6 months were grouped together as these categories have been shown to demonstrate equivalent response (Robertson et al, 1997). At relapse with bone metastases, all patients were fully staged radiologically and by liver function tests. Subsequently they were started on tamoxifen 20 mg day$^{-1}$ ($n$ = 21), megesterol acetate 160 mg day$^{-1}$ ($n$ = 5), Zoladex ($n$ = 2), aminoglutethamide ($n$ = 3), arimidex ($n$ = 1) and chemotherapy ($n$ = 1). Three women had been on adjuvant tamoxifen for over 5 years, and it was felt they might respond to tamoxifen withdrawal, thus they were given no treatment. No patient had received bisphosphonate therapy or skeletal radiotherapy prior to, or during, the study period. The study was approved by the Hospital Ethics Committee and all patients gave informed consent.

### Methods

Blood samples were collected at each clinic visit, centrifuged, and stored at –20°C. Urine for measurement of Pyr, Dpyr, calcium, creatinine and cyclic adenosine monophosphate (cAMP) was collected between 10:00 h and 12:00 h, from patients who had fasted overnight. Urine was stored at –20°C after the addition of 3% hydrochloric acid. All samples were coded, and all samples from individual patients were assayed in the same batch. Following acid hydrolysis of urine at 110°C overnight, and partial purification on a CF1 cellulose column, Pyr and Dpyr were separated by reverse phase high-performance liquid chromatography (HPLC) and detected by fluorescence (Colwell et al, 1993). The intra-assay coefficients of variation for Pyr and Dpyr were 7% and 10% respectively, and for inter-assay variation were <10%. Age-matched post-menopausal women had absolute ranges of Pyr of 12–40 nmol mmol$^{-1}$ creatinine, and Pyr 3–11 nmol mmol$^{-1}$ creatinine (Colwell et al, 1993). The upper limits of normal for this study were therefore the highest levels measured in age-matched controls; Pyr 40 nmol mmol$^{-1}$ creatinine and Dpyr 11 nmol mmol$^{-1}$ creatinine.

Urine excretion of cAMP was measured by radioimmunoassay (Amersham UK), and was expressed as nmol l$^{-1}$ glomerular filtrate. The fasting molar ratio of urinary calcium to creatinine (CaCr$^{-1}$ mmol mmol$^{-1}$) was multiplied by the serum creatinine to calculate calcium excretion (CaE). CaE was then compared with serum calcium to assess the renal tubular reabsorption of calcium (Peacock et al, 1969).

Serum calcium, creatinine, albumin, and alkaline phosphatase were measured by standard automated methods. Serum calcium was adjusted to an albumin concentration of 40 g l$^{-1}$ (adjusted serum calcium = measured calcium mmol l$^{-1}$ +0.02[40-measured albumin]). Blood for assay of plasma PTHrP was collected in EDTA after the addition of 200 IU of Trasylol, and separated by

**Table 1**

| | Responders | Progressors |
|---|---|---|
| Age | 56 years | 58 years |
| (range) | (42–69) | (36–82) |
| Disease-free interval from primary treatment | 51 months (0–276) | 12 months (0–168) |
| Number of initia bone metastases | 3 (1–12) | 5 (1–12) |
| Type of metastases | | |
| Lytic | 6 | 12 |
| Mixed | 11 | 7 |
| Other metastases | | |
| Soft tissue | 5 | 3 |
| Lung | 2 | 5 |
| Liver | 1 | 1 |
| Brain | 0 | 1 |
| Previous treatment[a] | | |
| None | 10 | 9 |
| Tamoxifen | 7 | 6 |
| Megace | 0 | 3 |
| DXT ovarian ablation | 0 | 1 |
| Treatment | | |
| None (withdrawal) | 2 | 1 |
| Tamoxifen | 10 | 11 |
| Megace | 3 | 2 |
| Aminoglutethamide | 1 | 2 |
| Zoladex | 1 | 1 |
| Arimidex | 0 | 1 |
| Chemotherapy | 0 | 1 |

[a] This includes 13 patients already on endocrine treatment for bone metastases, and 3 for soft tissue disease.

**Table 2**

| | Responders | Progressors | Range |
|---|---|---|---|
| $n$ | 17 | 19 | |
| Serum calcium | 2.29 | 2.32 | 2.1–2.6 |
| mmol l$^{-1}$ | (2.05–2.54) | (2.08–2.56) | NS |
| Urine calcium–creatinine ratio | 0.38 | 0.46 | <0.5 |
| | (0.01–0.99) | (0.05–1.24) | NS |
| Calcium excretion | 38 | 49 | |
| μmol LGF$^{-1}$ | (1–102) | (7–118) | NS |
| Urine cAMP | 36 | 61 | <60 |
| nmol LGF$^{-1}$ | (6–86) | (24–126) | $P$ < 0.05) |
| Pyr | 56 | 164 | <40 |
| nmol mmol$^{-1}$ creatinine | (20–75) | (35–291) | $P$ < 0.01 |
| Dpyr | 14 | 40 | <11 |
| nmol mmol$^{-1}$ creatinine | (6–22) | (9–80) | $P$ < 0.02 |
| PTHrP | <0.23 | <0.23 | <0.23 |
| pmol l$^{-1}$ | (<0.23–0.76) | (<0.23–0.65) | NS |
| CA 15-3 | 99 | 210 | <30 |
| IU | (11–498) | (29–853) | $P$ < 0.05 |

*© 1999 Cancer Research Campaign*

**Table 3**

| Marker for responders | Upper 95% CI prediction | Sensitivity | Specificity | Positive prediction |
|---|---|---|---|---|
| CA 15-3 IU $l^{-1}$ | >60 | 80% | 77% | 78% |
| Pyr nmol $mmol^{-1}$ creatinine | >70 | 80% | 93% | 93% |
| Dpyr nmol $mmol^{-1}$ creatinine | >22 | 84% | 95% | 95% |

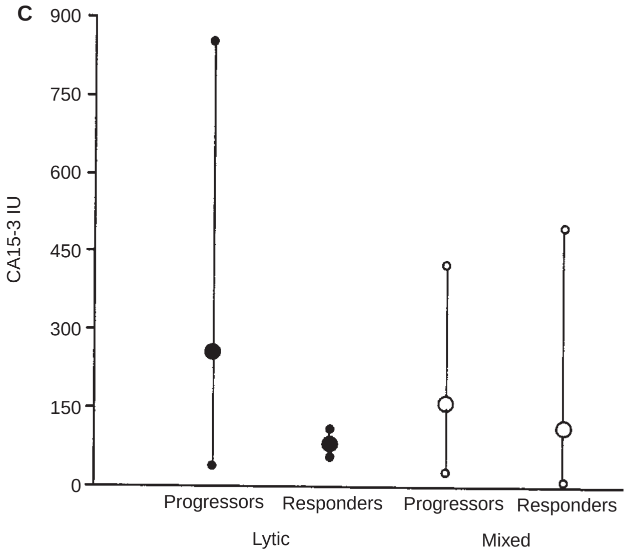

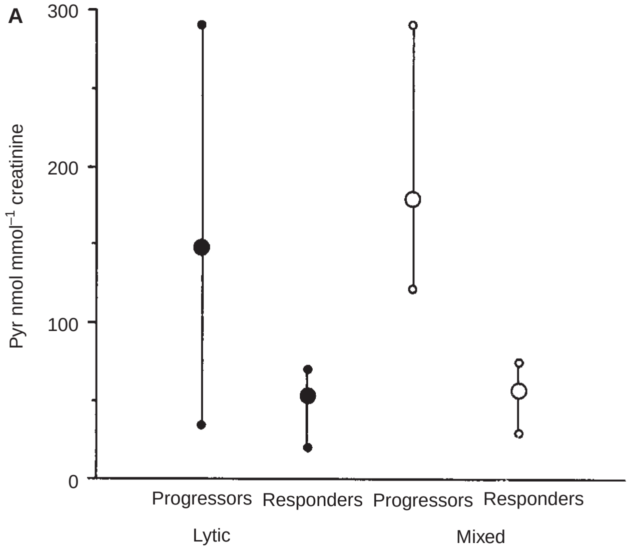

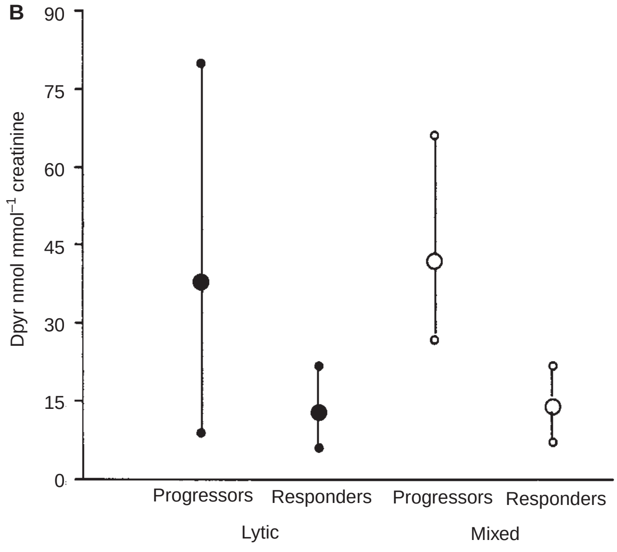

**Figure 1** (**A**) Initial urinary excretion of pyridinoline (Pyr) in progressors and responders, in patients with purely lytic, or mixed lytic/sclerotic bone metastases (data shown as absolute range and mean values). (**B**) Initial urinary excretion of deoxypyridinoline (Dpyr) in progressors and responders, in patients with purely lytic, or mixed lytic/sclerotic bone metastases (data shown as absolute range and mean values). (**C**) Initial serum CA15-3 in progressors and responders, in patients with purely lytic, or mixed lytic/sclerotic bone metastases (data shown as absolute range and mean values)

centrifugation within 15 min. Plasma PTHrP (1–86) was measured by a sensitive two-site immunoradiometric assay (normocalcaemic controls <0.23 pmol $l^{-1}$) (Ratcliffe et al, 1993). Serum CA15-3 was measured by an immunoradiometric assay (CIS UK Ltd; healthy controls >30 U $ml^{-1}$).

The percentage change of biochemical markers was calculated by using each patient as her own control. The subsequent increase or decrease in each tumour marker are expressed as a percentage change from the original value. This enables a comparison of magnitude of change between each tumour marker without any confusion as to the importance of absolute values. Variations of tumour markers within the reference range for healthy controls were recorded as no percentage change.

Statistical analysis was performed by Spearman rank correlation, Kruskal–Wallis three-way analysis, Mann–Whitney *U*-tests, and multiple analysis of variance where appropriate.

### RESULTS

### UICC assessment of disease response

The frequency of metastases in sites other than bone was equally distributed between patients with responding and progressing disease (Table 1). As evaluated by UICC criteria at 6 months, or at disease progression, 19 patients had radiological evidence of progressive disease, and 17 patients either had stable disease or a partial response. The median duration of response was 10 months (range 6–24 months). The median number of bone metastases initially detected in the women with progressive bone disease was five (range 1–12), and was not significantly different from the number found in women whose disease responded (median three, range 1–12). Eighteen women had lytic bone metastases, 14 had

*© 1999 Cancer Research Campaign* 

mixed sclerotic/lytic metastases, and four had sclerotic metastases with easily evaluable soft tissue metastases.

Measurement of urinary cross-link excretion at the start of the study showed that Pyr correlated with Dpyr ($r = 0.85$, $P < 0.0001$).

The number of bone metastases was not related to the urinary levels of the two bone resorption markers and there was no significant relationship between them and the number of bone metastases at presentation. The frequency of metastases in sites other than bone was equally distributed between the progressive and non-progressive groups (Table 1).

### Biochemical parameters at the start of the study

A series of urine and biochemical measurements were made at the beginning of the study in order to see if any measurement predicted disease response (Table 2). Excretion of Pyr was significantly higher in patients whose disease subsequently progressed (mean 164 nmol $mmol^{-1}$ creatinine, range 35–291) compared with a mean of 56 nmol $mmol^{-1}$ creatinine (range 20–75) in women who responded (partial response and stable disease) ($P < 0.01$) (Figure 1A).

Excretion of Dpyr was also higher in women with disease that progressed, mean 40 nmol $mmol^{-1}$ creatinine (range 9–80) compared with a mean value in responders of 14 nmol $mmol^{-1}$ creatinine (range 6–22) ($P < 0.02$) (Figure 1B). Tumour marker serum CA15-3 was also significantly higher in progressors (mean 210 IU, range 29–853) compared with responders (mean 99 IU, range 11–498) (Figure 1C) ($P < 0.05$). Serum CEA was also higher in progressors (mean 57, range 4–470) than in responders (mean 23, range 4–150) ($P < 0.05$).

Serum calcium, urine calcium/creatinine ratio, calcium excretion and plasma PTHrP were non-predictive (Table 2). Although urine cAMP was significantly higher at the beginning of the study in progressors, sequential changes were not predictive (Table 2). The specificity and sensitivity of three predictive assays (Pyr, Dpyr and CA15-3) are shown in Table 3.

### Patients with responding bone metastases

Pyr and Dpyr excretion was within the normal range in 50% and 60% of cases respectively. Furthermore, the urinary concentrations of Pyr and Dpyr did not change significantly throughout the period of urine cross-link sampling, which was for a median of 6 months (range 3–12), and demonstrated for the first 2 months by Figure 2A and B.

### Patients with progressive bone metastases

At initial diagnosis of skeletal metastases, urinary Pyr and Dpyr were increased in 100% and 85% of patients, respectively, who subsequently developed progressive disease (Figure 1A,B). The median follow-up time for a patient with progressive disease was 4 months (range 2–11). Over the period of monitoring, urinary Pyr and Dpyr excretion increased in all patients with progressive disease, compared with the levels at presentation. The initial rise in excretion preceded radiological evidence of disease progression by a median of 2 months (Figure 2A,B). The increases in Pyr and Dpyr excretion were significant at 8 weeks ($P < 0.04$), and also at 12 weeks ($P < 0.005$) (Figure 2A,B).

### Calcium excretion and PTHrP levels

Urinary calcium excretion at presentation did not differ significantly between the patients who progressed or responded; mean 49 μmol $LGF^{-1}$ (range 7–118) and 38 μmol $LGF^{-1}$ (range 1–102) respectively. Neither calcium excretion nor the urinary calcium–creatinine ratio correlated with disease response. Renal tubular reabsorption of calcium was within the normal range for all patients. Plasma PTHrP levels were similar in both groups of patients: the median in the progressive group was <0.23 pmol $l^{-1}$ (range <0.23–0.65) and for the non-progressive group was <0.23 pmol $l^{-1}$ (range <0.23–0.76).

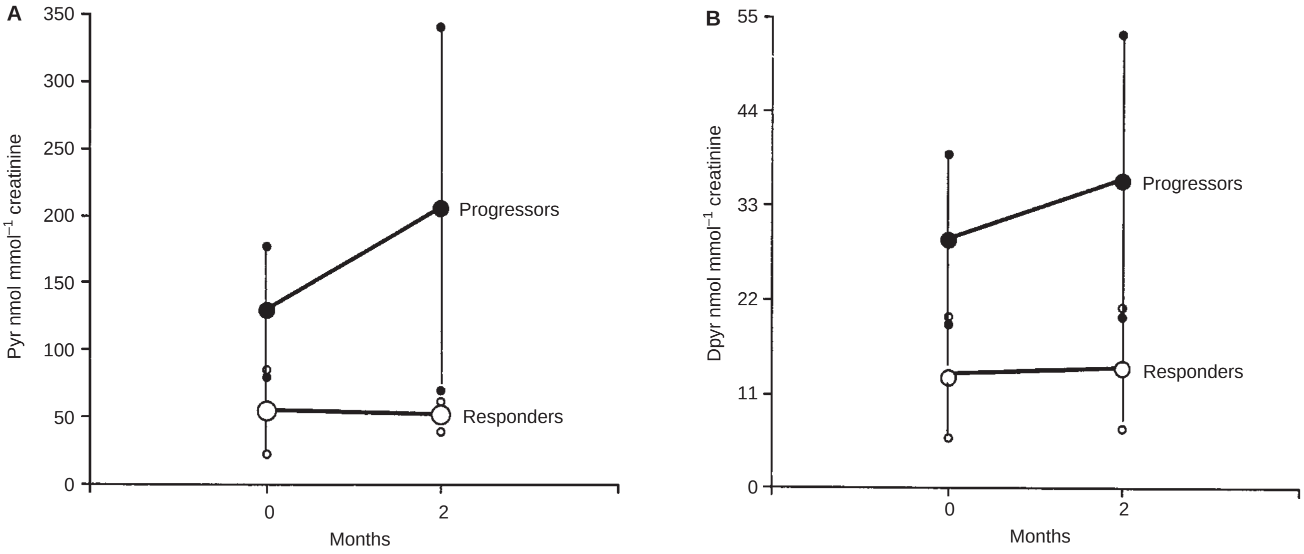

**Figure 2** (**A**) Changes in Pyridinoline (Pyr) excretion in progressors and responders, over the first two months of the study period (data shown as median values and standard deviation) (**B**) Changes in Deoxypyridinoline (Dpyr) excretion in progressors and responders, over the first two months of the study period (data shown as median values and standard deviation). Note: patients with progressive disease had further increase in pyridinoline and deoxypyridinoline

 © 1999 Cancer Research Campaign

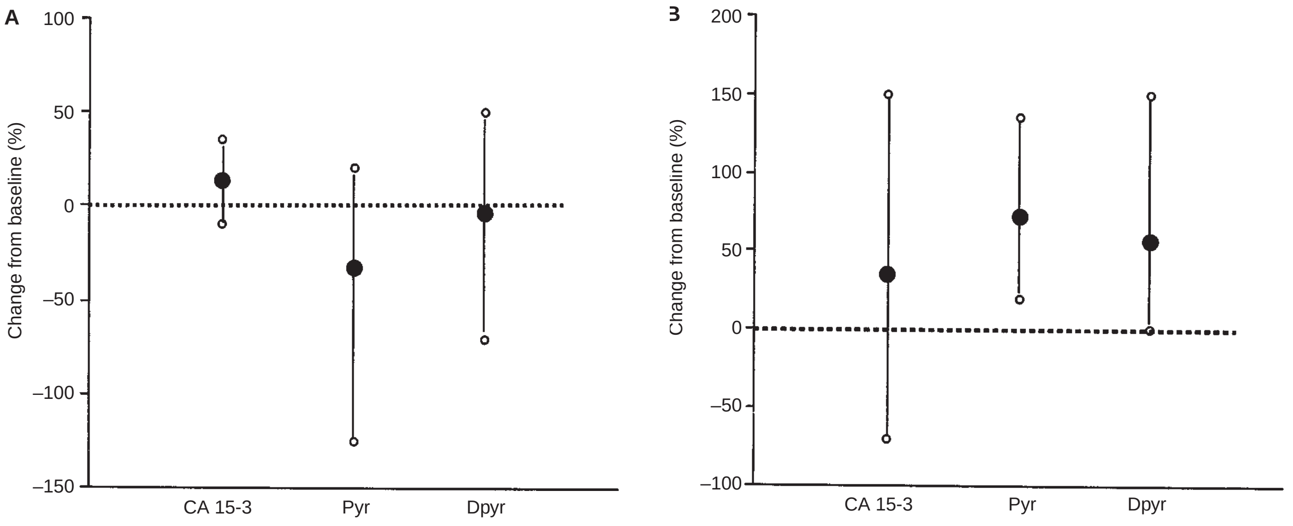

**Figure 3** (**A**) Percentage change in urinary excretion of Pyr and Dpyr, and serum CA15-3 over the first 2-month period of observation in patients who subsequently remained stable or responded to treatment. Patients who remained within the reference ranges/normal limits are represented as no percentage change. Results are expressed as absolute range and mean change. (**B**) Percentage change in urinary excretion of Pyr and Dpyr, and serum CA15-3 over the first 2-month period of observation in patients who subsequently progressed on treatment. Patients who remained within the reference ranges/normal limits are represented as no percentage change. Results are expressed as absolute range and mean change

### Comparison with changes in serum CA15-3 levels

In all patients, initial urinary Pyr and Dpyr levels correlated with serum CA15-3 ($r$ = 0.86, $P$ = 0.006; and $r$ = 0.8, $P$ = 0.01 respectively). In the group who progressed, median CA15-3 levels rose, and in the responders, median CA15-3 levels remained stable confirming the radiological UICC assessment of disease progression. However, CA15-3 levels increase from the first measurement in some responders, and decreased in some progressors (Figure 3A,B).

### DISCUSSION

The recent development of sensitive biochemical markers to determine changes in bone metabolism has provided new insights into the mechanisms of progression of bone metastases in women with breast cancer. Pyr and Dpyr have both been shown to be more sensitive than urinary hydroxyproline in monitoring bone resorption (Eastell et al, 1990; Bettica et al, 1992; Colwell et al, 1993).

We have confirmed the preliminary findings of a recent study which reported increased Pyr and Dpyr in patients with bone metastases from breast cancer (Paterson et al, 1991). Dpyr has previously been shown to decrease transiently in 20 patients with bone metastases undergoing chemotherapy, suggesting that Dpyr may be a useful early marker of disease response to therapy (Wada et al, 1993). We have demonstrated that the initial urine levels of Pyr and Dpyr in patients presenting with bone metastases allows prediction of future outcome of the disease. The positive predictive values that indicated the disease progression for Pyr and Dpyr were 95% and 100% for urine levels of 70 and 22 nmol $mmol^{-1}$ creatinine respectively. This compares very favourably with the standard tumour marker Ca15-3, where even with levels twice the upper limit of normal, sensitivity and specificity for disease progression was only 82% and 79%. Both Pyr and Dpyr increased significantly within 8 weeks in progressors. The measurement of urinary cross-links provided objective disease response assessment by 2 months compared to the standard criteria, which require 3–6 months of radiological assessment (Hayward et al, 1977; Coleman et al, 1998). Biochemical markers would thus allow earlier changes of therapy for women with progressive disease and may result in better patient outcomes.

CA 15-3 is released from human tumour cells, and is the most reliable biochemical tumour marker currently available for monitoring response to treatment in patients with advanced breast cancer. Although increased levels of CA 15-3 are useful, rises in CA 15-3 can occur in the first 3 months in some patients who subsequently respond to therapy, and should be regarded with caution. In contrast, Pyr and Dpyr are released from the bone matrix by osteoclast-mediated resorption stimulated by the tumour-produced factors. CA 15-3 is not produced by every tumour and does not always reliably increase even when the tumour is progressing (Robertson et al, 1991). However, CA 15-3 is of great value for monitoring metastases at other sites. Women who present with elevated urinary levels of cross-links appear to respond poorly to changes in hormonal therapy.

Breast cancer may affect bone metabolism, producing increased bone resorption with or without an associated increase in bone formation (Delmas et al, 1991; Sano et al, 1994). Bone resorption is increased by factors released by breast cancer cells such as PTHrP, TGF-$\alpha$, or prostaglandins, which may stimulate osteoclastic activity either locally or via the bloodstream (Mundy et al, 1985). Bone metastases usually stimulate bone resorption more than bone formation and thus markers of bone formation, e.g. alkaline phosphatase, are not as valuable markers of disease activity as markers of bone degradation such as Pyr and Dpyr.

Urinary cAMP excretion was raised at presentation in those patients who subsequently had progressive disease. It did not, however, rise sequentially with disease progression, and could not be utilized as a marker of disease response. Urinary cAMP was elevated in the absence of raised PTHrP, and this may reflect the

*© 1999 Cancer Research Campaign*

actions of an as yet unidentified tumour product that affects the kidney.

In contrast to previous authors (Coleman et al, 1988; Dodwell et al, 1990) we were unable to demonstrate a significant increase in urinary calcium excretion in patients with progressive bone disease. Urinary calcium excretion does not change in the presence of sclerotic bone metastases, whereas urinary cross-link excretion was shown to be elevated (Sano et al, 1994). In this study, 50% of the women had an element of sclerotic bone disease, and this may account for the failure of urinary calcium excretion to increase with disease progression. As with other markers of bone disease, elevation of urinary cross-link levels must be related to other clinical and biochemical markers as elevations of urinary cross-links could occur from benign bone disease. These results confirm work by Sano et al (1994) that bone resorption is also elevated in patients with osteosclerotic bone metastases despite the osteoblastic overactivity seen on histological and radiological study of such metastases. This study has demonstrated that sequential monitoring of urinary cross-link levels during therapy may prove to be a useful indicator of disease response in bone.

### REFERENCES

Bettica P, Moro L, Robins SP, Taylor J, Singer FR and Baylink DJ (1992) Bone-resorption markers galactosyl hydroxylysine, pyridinium crosslinks, and hydroxyproline compared. *Clin Chem* **38**: 2313–3218

Coleman RE and Rubens RD (1987) The clinical course of bone metastases from breast cancer. *Br J Cancer* **55**: 61–66

Coleman RE, Whittaker KB, Moss DW, Mashiter G, Fogelman I and Rubens RD (1988) Biochemical prediction of response of bone metastases to treatment. *Br J Cancer* **58**: 205–210

Coleman RE, Houston S, James I, Rodger A, Rubens RD, Leonard REF and Ford J (1992) Preliminary results of the use of urinary secretion of pyridinium cross links for monitoring metastatic bone disease. *Br J Cancer* **65**: 766–768

Colwell A, Russell RGG and Eastell R (1993) Factors affecting the assay of urinary 3-hydroxy pyridinium crosslinks of collagen as markers of bone resorption. *Eur J Clin Invest* **23**: 341–349

Delmas PD, Schlemmer A, Gineyts E, Riis B and Christiansen C (1991) Urinary excretion of pyridinium crosslinks correlates with bone turnover measured on iliac crest biopsy in patients with vertebral osteoporosis. *J Bone Min Res* **6**: 639–644

Dodwell DJ, Howell A and Ford J (1990) Reduction in calcium excretion in women with breast cancer and bone metastases using the oral bisphosphonate pamidronate. *Br J Cancer* **61**: 213–215

Eastell R, Hampton L, Colwell A and Reeve J (1997) Biochemical markers of bone resorption compared with estimates of bone resorption from radiotracer kinetics studies in osteoporosis. *JBMR* **12**: 59–65

Eyre DR (1990) Editorial: New biomarkers of bone resorption. *J Clin Endocrinol Metab* **74**: 470A–470C

Fujimoto D (1980) Evidence for natural existence and pyridinoline crosslink in collagen. *Biochem Res Commun* **93**: 948–953

Hayward JL, Carbone PP, Henson JC, Kumaoka S, Segaloff A and Rubens RD (1977) Assessment of response to therapy in advanced breast cancer. *Cancer* **39**: 1289–1293

Moro L, Gazzarrini C, Crivellari D, Galligioni E, Talamini R and de Bernard B (1993) Biochemical markers for detecting bone metastases in patients with breast cancer. *Clin Chem* **39**: 131–134

Mundy GR, Ibbotsin KJ and D'Souza SM (1985) Tumour products and the hypercalcaemia of malignancy. *J Clin Invest* **76**: 391–394

Paterson CR, Robins SP, Horobin JM, Preece PE and Cuschieri A (1991) Pyridinium crosslinks as markers of bone resorption in patients with breast cancer. *Br J Cancer* **64**: 884–886

Peacock M, Robertson WG and Nordin BEC (1969) Relation between serum and urinary calcium with particular reference to parathyroid activity. *Lancet* **i**: 384–386

Ratcliffe WA, Hutchensson ACJ, Bundred NJ and Ratcliffe JG (1992) Role of assays for parathyroid hormone-related protein in investigation of hypercalcaemia. *Lancet* **339**: 164–167

Robertson JF (1997) Letter. *Eur J Cancer* **33**: 1774–1779

Robertson JF, Pearson D, Price MR, Selby C, Blamey RW and Howell A (1991) Objective measurement of therapeutic response in breast cancer using tumour markers. *Br J Cancer* **64**: 757–763

Robins SP (1982) Turnover and crosslinking in collagen. In: *Collagen in Health and Disease*, Weiss JB and Jayson MIV (eds), pp. 160. Churchill Livingstone: Edinburgh

Sano M, Kyshida K, Takahashi M, Ohishi T, Okada M and Inoue T (1994) Urinary pyridinoline and deoxypyridinoline in prostate carcinoma patients with bone metastasis. *Br J Cancer* **70**: 701–703

Wada S, Katayama Y, Yasutomo Y, Kugai N and Nagata N (1993). Changes of bone metabolic markers in patients with bone metastases: clinical significance in assessing bone response to chemotherapy. *Intern Med* **32**: 611–618

 © 1999 Cancer Research Campaign
